# The era of digital mental health interventions: we know they can be effective but are they also safe?

**DOI:** 10.1192/bjo.2025.42

**Published:** 2025-04-21

**Authors:** Urska Arnautovska, Alyssa Milton

**Affiliations:** Health, Medicine and Behavioural Sciences, The University of Queensland, Brisbane, QLD, Australia; Metro South Addiction and Mental Health Service, Brisbane, QLD, Australia; Queensland Centre for Mental Health Research, Wacol, QLD, Australia; Faculty of Medicine and Health, The University of Sydney, Sydney, NSW, Australia; Australian Research Council Centre of Excellence for Children and Families over the Life Course, Sydney, NSW, Australia

**Keywords:** Adverse events, digital interventions, DMHI development, mental health services, safety

## Abstract

Over the past two decades, digital mental health interventions (DMHIs) have seen a surge in studies with people experiencing mental ill-health, whether this be via web-based platforms, smartphone applications, text messages or other digital devices. Although DMHIs already demonstrate evidence of their acceptability and some of their effectiveness among different populations, the information about their safety is less clear. This Editorial reflects on a Delphi study by Taher and colleagues that explored the regulation of DMHIs and generated ten safety recommendations. We discuss these recommendations in the context of existing relevant literature and provide suggestions for further steps to advance research and policy on DMHIs in the UK and globally. Further dialog is needed, including the views and experiences of all key stakeholders, and particularly of people with lived experience, to ensure DMHIs are not only an acceptable and potentially effective treatment approach, but also safe for those that use them.


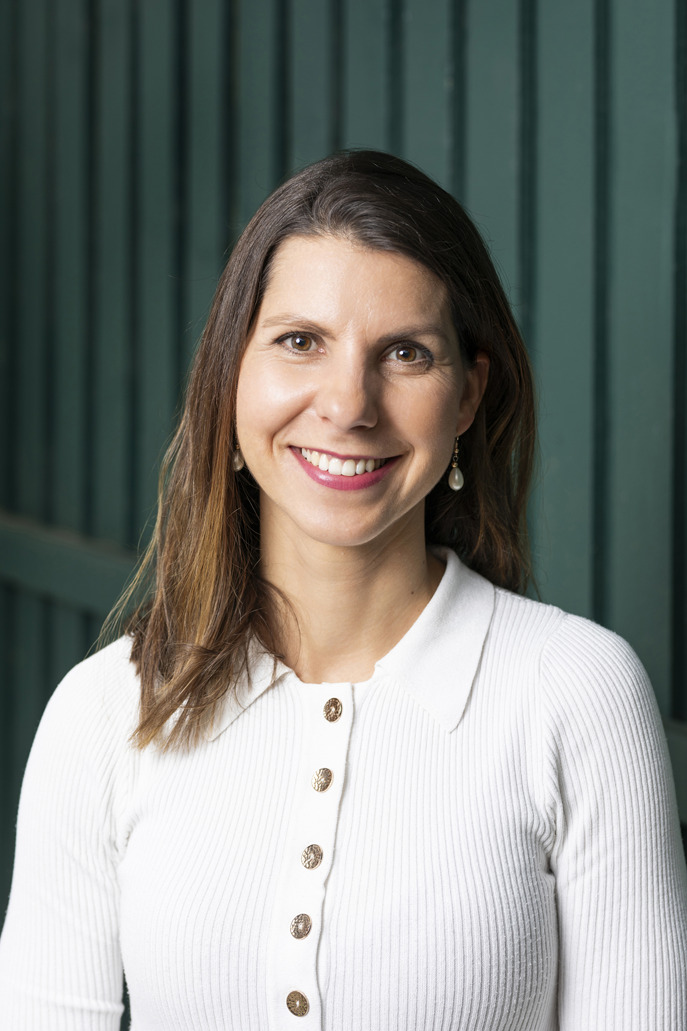
Over the past two decades, we have witnessed a rapid increase in the number of studies using digital mental health interventions (DMHIs), delivered remotely via computers, smartphone applications (apps), text messages or other digital devices such as wearables, aimed at improving various mental health conditions.^1^ Only a decade ago, the acceptability of DMHIs among populations experiencing mental ill-health was still unclear, but systematic reviews^2^ and individual empirical studies published since then have provided strong evidence of DMHIs being an acceptable and potentially effective treatment option for people with mental ill-health across a range of subpopulations.^3^

There is no doubt that such approaches can overcome some of the main limitations of traditional, face-to-face mental health treatments, such as costs associated with face-to-face delivery, restricted access and reach (often tied to clinical settings), along with challenges in supporting continuity of care during critical transition points (e.g. discharge from in-patient treatment).^4^ However, it is currently less clear if DMHIs present a safe treatment option. The safety of DMHIs, and psychological interventions in general, is typically defined by the occurrence of unwanted effects or negative outcomes, categorised as adverse events or serious adverse events (SAEs). SAEs, which include outcomes like death, disability or hospital admission, are consistently defined because of strict regulatory frameworks. In contrast, there is greater variability in conceptualising and measuring non-SAE, encompassing a broad range of less severe negative effects such as psychological harm (e.g. distress caused by content) and exacerbation of symptoms (e.g. worsening of anxiety or depression). An additional consideration of safety includes risks related to data privacy and security, which are critical in DMHIs, particularly when dealing with sensitive information or in high-risk scenarios, such as suicide prevention apps, where a trade-off between monitoring for safety and ensuring user privacy must be carefully balanced.

One reason for the lack of clarity around the safety of DMHIs is likely related to the fact that, although a small proportion of DMHIs have been tested rigorously in sufficiently powered randomised clinical trials^5^ (e.g. the Horyzons trial:^6^ *n* = 170, found no significant differences compared with treatment as usual in the primary outcome (social functioning), but positive trends in vocational outcomes and use of emergency services; and the EMPOWER trial:^7^ *n* = 73, found lower fear of relapse in the EMPOWER group compared with treatment as usual), most DMHIs are available freely on the App store and lack proper evidence of safety and efficacy. As such, the issue is two-fold: first, there is a largely unregulated market supporting the existence of DMHIs, and second, we currently lack a regulatory method that is appropriate for the assessment of DMHIs. Currently, DMHIs are typically classified as ‘medical devices’ and, therefore, are considered to fall under regulatory processes that are responsible for regulating both pharmaceuticals and medical devices (e.g. the Medicines and Healthcare products Regulatory Agency in the UK, Therapeutic Goods Administration in Australia and Digital Services Act in the USA). Merging DMHIs with traditional medical interventions, however, risks overlooking critical aspects specific to mental health contexts.

To address this risk, a recent Delphi study by Taher and colleagues^8^ involved an international panel of 20 research experts suggesting solutions on how to adapt medical regulatory methods to better suit DMHIs. The strength of this research is in pursuing a regulatory solution that would be tailored to mental health interventions delivered using various technology platforms, given that DMHIs are radically different compared with pharmaceutical interventions, and are characterised by the large heterogeneity of digital approaches and their common use as adjuncts to care, rather than as standalone treatments. Valuable outcomes of the Delphi study are a list of ten safety recommendations that reached consensus and the implementation strategies to support the assessment of the safety of DMHIs (Table 1).

**Table 1:** Recommendations on how to improve the safety of digital mental health interventions and strategies for their implementation

Theme	Ten recommendations that reached consensus	Strategies to implement recommendations
Improving adverse events data in DMHI trials	Publish guidelines for professionals on how to assess and report adverse event data in digital mental health research	Generate publication guidelines for reporting adverse events data that are required by journals to ensure that adverse event data are collected and reported consistently These guidelines should outline what adverse events data should be collected, when and how this should happen, and how these should be categorised, analysed and reported Determine the relatedness of the adverse event to the therapy
	Standardise the optimum way to assess relatedness of an adverse event to the intervention, procedure or device, by using a variety of independent evidence sources	
	When evaluating safety, differentiate between self-guided and clinician guided interventions	
Broadening the definition of serious adverse events for DMHIs	Broaden the scope of potential serious adverse events in digital studies to consider impact on mental health	The current definition of serious adverse events (hospitalisation, death, congenital abnormalities…) should be extended to be inclusive of: Serious effects on mood, behaviour, general well-being and functioning, not just physical harms Increase in harm toward others Increase in intentional self-harm Seizures induced by on-screen flashes A suicide attempt
Short-term deterioration is not a safety concern in mental health therapy	Monitor psychological deterioration to prevent potential harm, using post-treatment follow-ups	Develop and maintain a strong therapeutic alliance Discuss a clear rationale for each therapy activity/exercise Patients need to be informed about short-term symptom deterioration/distress (through the process of informed consent). In a digital context this can be replicated through text, audio or video content It is important for therapists to normalise symptom deterioration. This can happen as part of a discussion with a trained mental health professional, or via the content of the digital therapy Help the patient think about sources of support that they can use between sessions if needed. In a digital context, provide users with alternative sources of support should they need it Provide patients/users with information on how to manage symptom deterioration
	Utilise the suggestions outlined to support patients experiencing deterioration in any treatment context	
Adapting the yellow card scheme for DMHIs	Improve the visibility of the yellow card scheme as the primary means of reporting adverse reactions in the digital mental health field	Raise awareness among users that the yellow card system is used for reporting adverse reactions in digital mental health therapies Use a broader category such as ‘mental health adverse events’ is more appropriate. Include: Severity/impact on the individual Suicidal ideation/behaviour Intentional self-harm Threat to others
	Change the yellow card ‘psychiatric conditions’ category label to ‘mental health adverse reactions’	
	Expand the yellow card category that captures mental health data to incorporate informative subcategories and additional contextual information	
		Patient-reported outcomes automatically collected within the digital therapy itself
Using a harmonised approach to assess the safety of psychological therapies generally	Develop an overarching, harmonised framework for assessing the safety of any psychological intervention (digital or otherwise)	Have a harmonised approach for safety reporting across face-to-face and digital interventions to increase our knowledge of the risks of mental health therapies and contribute to stronger safety standards and safer interventions

DMHI, digital mental health intervention.

Reflecting on these recommendations, our own experience with co-production and delivery of DMHIs,^4^ as well as previous initiatives aiming to capture the strengths and weaknesses regarding the methodology and safety of DMHIs,^9^ we point out several key elements and steps to advance this research and policy in this field. First, the implementation of a formalised regulatory method is essential for ensuring that DMHIs are both effective and safe, and do not inadvertently increase the risks of harm to their users, ultimately benefiting a broader range of individuals. Second, the safety of DMHIs should be assessed early in pilot feasibility studies, addressing any issues raised by key end users before scaling up. Involving end users throughout the development process of DMHIs early may also improve the implementation of digital treatment approaches, including user engagement with DMHIs, which, in turn, is likely to facilitate positive outcomes.^10^ Third, the need for assessment of the safety of DMHIs early in the research process underscores the importance of regulatory boards such as human research ethics committees being familiar with the guidelines for evaluation of DMHIs, such as the World Health Organization mHealth Evaluation, Reporting and Assessment (mERA) checklist.^11^ Such formal regulatory processes would ensure that any risks associated with a specific DMHI to be used in a subsequent study, along with detection and mitigation of these risks, are clearly outlined in the risk evaluation and management plan within a study/trial protocol. Finally, we underscore the need for involvement of people with lived experience, especially those using DMHIs, to tailor these guidelines to specific conditions and risks.

The latter point leads us to a key limitation of the Delphi study by Taher and colleagues: the lack of lived experience,^12^ service user/patient and carer perspectives and an overrepresentation of views from those working in higher education. It is essential that all key end users, including people with lived experience of a specific mental health condition as well as clinicians and other therapists, provide their perspectives on what aspects of safety should be monitored and possibly mitigated when using DMHIs within real-life clinical and community settings. As such, a concept mapping approach may be better suited to capture the perspectives of all of these diverse users.

An example of a successful use of the concept mapping methodology to generate best practice in mental health is the Crisis Resolution Team Optimisation and Relapse Prevention (CORE) study.^13^ Through involvement of multiple stakeholder groups who prioritised and grouped 72 statements derived from various soruces,^14^ their integrative approach enabled the development of a fidelity scale for crisis resolution teams, which was then piloted widely across UK,^15^ demonstrating feasibility and validity in its use. As such, using a concept mapping approach can ensure a more comprehensive understanding of what constitutes ‘safety’ from diverse perspectives and develop tailored, context-specific measures that reflect the unique features of DMHIs.

Further, involvement of people with lived experience is not only crucial during development, reporting and testing, but also in real-life implementation and monitoring of the intervention usage,^12^ which would provide further reassurance of their safety, or identify needs for further improvement of safety. Additionally, exploration of the role of peer workers/coaches in the delivery and assessment of safety of DMHIs may be an avenue explored further in future research. Adding these voices to the regulations around the safety of DMHIs may be especially important in the context of the increasing complexity of DMHIs, which may involve several end users (e.g. people experiencing mental ill-health, peer coaches, therapists, online peer community).

In line with this next step, it would also be important to include experts and end users from the Global South,^16^ as 85% of experts in the Taher et al study were based in the UK, with the remainder representing countries such as Canada, Australia and the Netherlands. Related to this point is also the need to make regulatory processes more accessible and affordable. As DMHIs developers, we are aware of the high costs and staffing demands associated with proper testing of such treatment approaches. Although national research funding bodies may fund such rigorous testing of a few lucky examples of DHMIs, to ensure the safety of a large majority of existing DMHIs, a different, stepped-level and potentially industry-driven, regulatory approach may be needed. Finally, it is essential to ensure that regulatory standards are proportionate and not unduly stringent for DMHIs, especially compared with pharmacological interventions. For pharmacological treatments, the risk of SAEs can be higher and conceptualised more clearly than in non-pharmacological trials. Although DMHIs do require high-quality safety monitoring, the potential for harm should not be overstated compared with pharmacological trials. Given that these two treatments options involve different risks, safety frameworks should reflect these differences to avoid creating an unfair imbalance in the quality standards between both treatment approaches.

In conclusion, it is evident that the issue of applying regulatory processes pertinent to pharmaceutical and medical devices to DMHIs warrants further dialog. To advance this dialog, considering many more views, including those using DMHIs, would be paramount. This may be especially critical, considering the fast-growing number of innovative DMHI that integrate artificial intelligence and related data collection methods (e.g. photos, posts on social medial platforms). Further, although implementing the recommendations from the Delphi study has the potential to enhance the safety assessment processes for DMHIs, embedding these recommendations into existing frameworks such as World Health Organization mERA^11^ checklist can present a useful step toward establishing formal regulatory methods for critical assessment and transparency in reporting of risks associated with digital technologies that aim to improve health outcomes across populations with mental ill-health. Improving the quality of mental health safety data, refining the risk mitigation processes and conducting more accurate risk–benefit analyses have the potential to lead to more effective and safer DMHIs, which, in the current digital era, have a strong potential to become a useful treatment partner across a wide range of mental health settings.

## References

[1] Torous J , Myrick K , Aguilera A. The need for a new generation of digital mental health tools to support more accessible, effective and equitable care. World Psychiatry 2023; 22: 1. 36640397 10.1002/wps.21058 PMC9840484

[2] Miralles I , Granell C , Díaz-Sanahuja L , Van Woensel W , Bretón-López J , Mira A , et al. Smartphone apps for the treatment of mental disorders: systematic review. JMIR mHealth uHealth 2020; 8: e14897. 32238332 10.2196/14897 PMC7163422

[3] Fisher M , Etter K , Murray A , Ghiasi N , LaCross K , Ramsay I , et al. The effects of remote cognitive training combined with a mobile app intervention on psychosis: double-blind randomized controlled trial. *J Med Internet Res* 2023; 25: e48634. 10.2196/48634 PMC10682932 37955951

[4] Milton A , Ozols AMI , Cassidy T , Jordan D , Brown E , Arnautovska U , et al. Co-production of a flexibly delivered relapse prevention tool to support self-management for long-term mental health conditions: a co-design and user-testing study. *JMIR Format Res* 2024; 8: e49110. 10.2196/49110 PMC10926903 38393768

[5] Arnautovska U , Trott M , Vitangcol KJ , Milton A , Brown E , Warren N , et al. Efficacy of user self-led and human-supported digital health interventions for people with schizophrenia: a systematic review and meta-analysis. Schizophr Bull [Epub ahead of print] 27 Sep 2024. Available from: 10.1093/schbul/sbae143. PMC12414553 39340312

[6] Alvarez-Jimenez M , Koval P , Schmaal L , Bendall S , O’Sullivan S , Cagliarini D , et al. The Horyzons project: a randomized controlled trial of a novel online social therapy to maintain treatment effects from specialist first-episode psychosis services. World Psychiatry 2021; 20: 233–43. 34002511 10.1002/wps.20858 PMC8129860

[7] Gumley AI , Bradstreet S , Ainsworth J , Allan S , Alvarez-Jimenez M , Aucott L , et al. The EMPOWER blended digital intervention for relapse prevention in schizophrenia: a feasibility cluster randomised controlled trial in Scotland and Australia. Lancet Psychiatry 2022; 9: 477–86. 35569503 10.1016/S2215-0366(22)00103-1

[8] Taher R , Bhanushali P , Allan S , Alvarez-Jimenez M , Bolton H , Dennison L , et al. Bridging the gap from medical to psychological safety assessment: consensus study in a digital mental health. BJPsych Open 2024; 20: e126. 10.1192/bjo.2024.713 PMC11363077 38828683

[9] Torous J , Firth J , Mueller N , Bendall S , Onnela JP , Baker JT. Methodology and reporting of mobile heath and smartphone application studies for schizophrenia. Harv Rev Psychiatry 2017; 25: 146. 28234658 10.1097/HRP.0000000000000133 PMC5419869

[10] Berry N , Machin M , Ainsworth J , Berry K , Edge D , Haddock G , et al. Developing a theory-informed smartphone app for early psychosis: learning points from a multidisciplinary collaboration. Front Psychiatry 2020; 11: 602861. 33362612 10.3389/fpsyt.2020.602861 PMC7758439

[11] Agarwal S , LeFevre AE , Lee J , L’engle K , Mehl G , Sinha C , et al. Guidelines for reporting of health interventions using mobile phones: mobile health (mHealth) evidence reporting and assessment (mERA) checklist. BMJ 2016; 352: i1174. 26988021 10.1136/bmj.i1174

[12] Palmer VJ , Banfield M. Omission of mental health lived-experience research in implementation research commission. Lancet Psychiatry 2024; 11: 409. 10.1016/S2215-0366(24)00114-7 38760107

[13] Lloyd-Evans B , Christoforou M , Osborn D , Ambler G , Marston L , Lamb D , et al. Crisis resolution teams for people experiencing mental health crises: the CORE mixed-methods research programme including two RCTs. Program Grants Appl Res 2019; 7: 1–102. 30986008

[14] Lloyd-Evans B , Bond GR , Ruud T , Ivanecka A , Gray R , Osborn D , et al. Development of a measure of model fidelity for mental health Crisis Resolution Teams. BMC Psychiatry 2016; 16: 1–12. 27905909 10.1186/s12888-016-1139-4 PMC5133753

[15] Lamb D , Milton A , Forsyth R , Lloyd-Evans B , Akther S , Fullarton K , et al. Implementation of a crisis resolution team service improvement programme: a qualitative study of the critical ingredients for success. Int J Ment Health Syst 2024; 18: 18. 38704589 10.1186/s13033-024-00638-6 PMC11069280

[16] Kumar M. Championing equity, empowerment, and transformational leadership in (mental health) research partnerships: aligning collaborative work with the global development agenda. Front Psychiatry 2019; 10: 421791. 10.3389/fpsyt.2019.00099 PMC6432896 30936839

